# Oxygen—Its Agency in Therapeutics

**Published:** 1886-12

**Authors:** B. M. Lawrence


### OXYGEN—ITS AGENCY IN THERAPEUTICS.

BY B. M. LAWRENCE, M. D.

### NO. I.

Every day brings forward some new theory with regard to the curing of disease, and, as the result, there is a continual change transpiring in the practice of medicine. The injurious and too often fatal consequences which followed the old school methods of dispensing large portions of dangerous poisons, so prevalent in former times when mercury or calomel was regarded as the Sampson of *materia medica*, created a demand for a new and milder school of medical treatment, and all modern improvements in therapeutics received a warm welcome from the people and the progressive physician.

One extreme uniformly leads to another. Paraselsus in due time paved the way for the advent of Hahnemann, and Allopathy became the legitimate father or forerunner of Homeopathy. Truth is usually found between the two extremes.

The remarkable success which frequently followed the administration of small doses of medicine in time gave birth to the metaphysical method of treatment, which entirely discards all manner of drugs ; and, already under the names of mind cure, mental healing, faith cure or Christian science—falsely so called—in some cities almost an anti-drug mania has been developed. The reaction which must surely follow embryotic phases of psychopathy will not fail to greatly increase the growing interest everywhere manifested of late in manipulations—massage, magnetism and electricity, as necessary auxiliaries and essential prerequisites in successfully treating a very large class of chronic and nervous diseases.

These facts and conclusions, taken in connection with the entire history of the art of healing, will enable the careful investigator to comprehend more fully—while tracing the footprints of evolution in medical practice—why the attention of both physicians and patients has recently become so uniformly and intimately interested in the employment of Oxygen and its compounds as agents in therapeutics.

When the conscientious doctor gradually loses confidence in his drugs, and reaches the conclusion arrived at by the most eminent medical authors, that his most popular remedies are deadly poisonous drugs, which destroy more human lives than "war, pestilence and famine" combined ; that each dose administered is only a "blind experiment upon the patient's vitality," and that to "throw physic to dogs," or to force it down their throats, would be gross "cruelty to animals," while throwing it into the sea would prove "fatal to the fishes," his attention is at once turned into some other channel in search of more rational remedies. As he loses confidence in the poisons which destroy life, he gains faith in all normal and harmless hygienic agencies.

Air, water, sunlight, food, clothing, rest, exercise, social relations, and all the conditions of harmony and health, now claim his careful attention. But with each of these he still fails to find a complete *materia medica* sufficient for all emergencies, and electricity as a factor in therapeutics may next come up for investigation. His knowledge of this most wonderful agent assures him that it is accomplishing truly marvelous results in nearly every other art or science, and therefore he asks why may it not do still greater things if properly employed as a remedy for the numerous "ills that flesh is heir to."

But the question arises, What is electricity? The enthusiastic electrician—who really believes that this mysterious and really valuable curative is an all-potent panacea for every human ill—will directly inform you that "Electricity is life," but he finds himself entirely unable to comprehend or explain what LIFE is; but one fact must be admitted by all—there can be neither electricity or life manifested in any form or under any circumstance without OXYGEN. This element is the most abundant active and powerful agent known in all of nature's vast domain. It is the primal force which sets in motion every current of electricity that flows along the countless miles of wires stretched all over the lands and under the deepest seas. Oxygen causes the chemical changes which manufacture or set in motion this wonderful fluid, and, with the speed of light, sends it onward to every city and hamlet in the civilized world. Oxygen is the subtile source of chemical affinity, and therefore the ever-active principle that charges the electro-magnetic machine which the electrical doctor or operator employs in sending the currents through the human system, and, when an atmosphere surcharged with oxygen is inhaled and absorbed by the blood, a chemical combination follows which *generates vitalized electricity* in every artery and capillary in all parts of the body, creating most agreeable, restful, and invigorating sensations.

The anatomist finds that each artery is followed through all its intricate ramifications by a nerve, which serves to take up by induction the nervo-vital fluid, which becomes the *vis medacatix natura*, or active living force which builds up, heals, sustains, governs and controls all the organs and their functions, actions and motions, voluntary and involuntary, giving tone, strength and vigor to both the mind and body.

Oxygen, therefore, may be regarded as the *Rex Magnus*, or great ruling element in every department of nature's most marvelous laboratory, the great creative agent of life in, by and through which "we live, move and have our being."

The continual change of structure, by means of the constant tearing down of the old and worn-out tissues, and simultaneous building up of a new growth, so essential to physical beauty and wealth, can only be accomplished by this great reconstructive, without which no vital or physical change can transpire. Oxygen is the essence of all life, animal or vegetable. Our bodies require and receive a constant supply of it from the air we breathe, the food we eat and the water we drink; its absence causes death, its deficiency some form of disease.

Early in the earth's history, we are assured, there was a geological era when the atmosphere lacked the due amount of oxygen required to support animal life, and the air in miasmatic localities is still deficient, or destitute of a full supply. Imperfectly ventilated dwellings, crowded halls, churches and theatres, are always deprived of the normal quantity of oxygen, on which a state of perfect health depends. Our lamps, gas jets, stoves and furnaces, take from the atmosphere we inhale a part of that "breath of life" which we are taught first made man a "living soul."

The slightest degree of compression about the vital organs, by means of closely-fitting apparel, such as is usually worn by most men as well as women, never fails to diminish to a greater or less extent the complete supply of this great invigorating and vitalizing element, thus laying the foundation for the fearful ravages of consumption and many other dreadful forms of disease.

From the foregoing facts—which will be readily admitted by all who have given sufficient attention to the science of caring for the body in health and disease—it remains no longer a difficult matter to understand the rationale of the comparatively new mode of curing disease by the inhalation of what is known as compound oxygen, which may be perhaps much more appropriately designated as "The Oxygen Treatment."

Some practical suggestions and clinical experience of special interest to the invalids and physicians, who may desire to know more about the *modus operandi* of oxygen gas as an agent in therapeutics, will be considered in the next number of this JOURNAL.

